# The effect of excess weight on circulating inflammatory cytokines in drug-naïve first-episode psychosis individuals

**DOI:** 10.1186/s12974-018-1096-6

**Published:** 2018-02-28

**Authors:** María Juncal-Ruiz, Laura Riesco-Dávila, Víctor Ortiz-García de la Foz, Mariluz Ramírez-Bonilla, Obdulia Martínez-García, Juan Irure-Ventura, Juan Carlos Leza, Marcos López-Hoyos, Benedicto Crespo-Facorro

**Affiliations:** 1 Department of Psychiatry, Sierrallana Hospital, IDIVAL, School of Medicine, University of Cantabria, Torrelavega, Spain; 2 Department of Psychiatry, Marqués de Valdecilla University Hospital, IDIVAL, School of Medicine, University of Cantabria, Santander, Spain; 3 Department of Immunology, Marqués de Valdecilla University Hospital, IDIVAL, School of Medicine, University of Cantabria, Santander, Spain; 4 Centro de investigación Biomédica en Red de Salud Mental (CIBERSAM), Santander, Spain; 5 Centro de Investigación Biomédica en Red de Salud Mental (CIBERSAM), Complutense University, Madrid, Spain; 6 Department of Pharmacology, Faculty of Medicine, Complutense University, Madrid, Spain; 7 Instituto de Investigación Sanitaria (IIS) Hospital 12 de Octubre (i+12), Madrid, Spain

**Keywords:** Psychosis, Body mass index, Low-grade inflammation, Cytokines, Chemokines

## Abstract

**Background:**

Low-grade inflammation has been repeatedly associated with both excess weight and psychosis. However, no previous studies have addressed the direct effect of body mass index (BMI) on basal serum cytokines in individuals with first-episode psychosis (FEP).

**Objectives:**

The aim of this study is to analyze the effect of BMI on basal serum cytokine levels in FEP patients and control subjects, separating the total sample into two groups: normal-weight and overweight individuals.

**Methods:**

This is a prospective and open-label study. We selected 75 FEP patients and 75 healthy controls with similar characteristics to patients according to the following variables: sex, age, and cannabis and tobacco consumption. Both controls and patients were separated into two groups according to their BMI: subjects with a BMI under 25 were considered as normal weight and those with a BMI equal to or more than 25 were considered as overweight. Serum levels of 21 cytokines/chemokines were measured at baseline using the Human High Sensitivity T Cell Magnetic Bead Panel protocol from the Milliplex® Map Kit. We compared the basal serum levels of the 21 cytokines between control and patient groups according to their BMI.

**Results:**

In the normal-weight group, IL-8 was the only cytokine that was higher in patients than in the control group (*p* = 0.001), whereas in the overweight group, serum levels of two pro-inflammatory cytokines (IL-6, *p* = 0.000; IL-1β, *p* = 0.003), two chemokines (IL-8, *p* = 0.001; MIP-1β, *p* = 0.001), four Th-1 and Th-2 cytokines (IL-13, *p* = 0.009; IL-2, *p* = 0.001; IL-7, *p* = 0.001; IL-12p70, *p* = 0.010), and one Type-3 cytokine (IL-23, *p* = 0.010) were higher in patients than in controls.

**Conclusions:**

Most differences in the basal serum cytokine levels between patients and healthy volunteers were found in the overweight group. These findings suggest that excess weight can alter the homeostasis of the immune system and therefore may have an additive pro-inflammatory effect on the one produced by psychosis in the central nervous system.

## Background

Inflammation is usually a physiological response of the organism to harmful stimuli, whether physical, chemical, or biological. The inflammatory state that accompanies excess weight shows a peculiar presentation, as it is not accompanied by infection or sign of autoimmunity and no massive tissue injury seems to occur. In fact, it is often called “low-grade” chronic inflammation [17]. Cytokines are hormone-like proteins that enable immune cells to communicate and play an integral role in the initiation, perpetuation, and subsequent down-regulation of the immune response [28]. Several studies have found that overweight individuals (body mass index (BMI) ***≥*** 25) have higher plasma levels of some pro-inflammatory cytokines such as IL-6, TNF-α, and IL-1β than normal-weight individuals [16, 22].

Obesity is reported in approximately 50% of patients with schizophrenia [2, 3]. In addition, a pro-inflammatory state has been repeatedly associated with first-episode psychosis (FEP) [5, 11, 12, 15], especially in women, overweight patients, and tobacco users [9, 10]. However, no previous study has addressed the direct effect of BMI on basal serum cytokines in FEP individuals.

The aim of this study is to analyze the effect of BMI on basal serum cytokine levels in FEP patients and control subjects, separating the total sample into two groups: normal-weight and overweight individuals. Our hypothesis is that higher levels of inflammatory cytokines will be observed in patients than in controls in the overweight group with respect to this same comparison in the normal-weight group.

## Methods

### Study setting

The data for the present study were obtained from a large epidemiological cohort of patients who have been treated in a longitudinal intervention program of FEP (PAFIP: *Programa de Atención a Fases Iniciales de Psicosis*) conducted at the University Hospital Marqués de Valdecilla in Cantabria, Spain. The main procedures that are carried out in this program have been described elsewhere [19]. The program was approved by the local institutional review board, and informed consent was obtained from patients and their families prior to inclusion.

### Subjects

In June 2016, 75 patients with FEP drug-naïve, who were previously included in PAFIP from June 2011 to May 2016, were selected to entry in this study according to the following criteria: (1) age between 15 and 50, (2) residency in the catchment area, (3) experiencing their first-episode of psychosis, (4) no prior treatment with antipsychotic medication, (5) DSM-IV criteria for brief psychotic disorder, schizophreniform disorder, schizophrenia, schizoaffective disorder, or psychotic disorder not otherwise specified. Patients were excluded if they met any of the following criteria: (1) DSM-IV criteria for drug dependence, (2) DSM-IV criteria for mental retardation, and (3) history of neurological disorder or brain injury. The diagnoses were confirmed by an experienced psychiatrist, applying the Structured Clinical Interview for DSM-IV (SCID-I) 6 months following the baseline visit.

### Study design

This is a prospective, open-label study. We selected 75 healthy volunteers with similar characteristics to patients according to some possible confounding variables, such as sex, age, and both cannabis and tobacco consumption, that might have a pro-inflammatory effect. Both controls and patients were separated into two groups according to their BMI: those subjects with a BMI under 25 were considered as normal-weight and those ones with a BMI equal or over 25 were considered as overweight. Fasting venous blood samples were collected at baseline.

We have performed four comparisons of the basal serum levels of the 21 cytokines between the control group and the patient group: (1) between normal-weight controls and normal-weight patients, (2) between overweight controls and overweight patients, (3) between normal-weight controls and overweight controls, and (4) between normal-weight patients and overweight patients.

### Serum cytokine/chemokine measurement

Serum cytokine/chemokine levels and different profiles were measured using the Human High Sensitivity T Cell Magnetic Bead Panel protocol from the Milliplex® Map Kit (cat. no. HSTCMAG28SPMX21, EMD Millipore, Billerica, MA 01821, USA), following the manufacturer’s instructions. Briefly, assay plates were washed with wash buffer, sealed, and mixed on a plate shaker for 10 min at room temperature. The wash buffer was decanted and 50 μL of the diluted standards, quality controls, and serum samples were added into the appropriate wells. After the addition of the samples or controls, the plates were incubated overnight at 4 °C on a plate shaker with fluorescently labeled capture antibody-coated beads. After overnight incubation with capture antibodies to detect Fractalkine, GM-CSF, IFNγ, IL-1β, IL-2, IL-4, IL-5, IL-6, IL-7, IL-8, IL-10, IL-12 (p70), IL-13, IL-17A, IL-21, IL-23, ITAC, MIP-1α, MIP-1β, MIP-3α, and TNF-α, well content was removed and washed using a handheld magnet. Then, 50 μL of biotinylated detection antibodies were added into each well and incubated for 1 h at room temperature while shaking. Following, without removing well content, 50 μL of streptavidin-phycoerythrin was added into each well. After incubation for 30 min at room temperature, the samples were washed using a handheld magnet and resuspended in sheath fluid. Finally, the samples were run on the Luminex 100/200 and the data were collected using the Luminex xPONENT® software (v. 3.1). Analysis of the cytokine/chemokine median fluorescence intensity (MFI) was performed using the MasterPlex® QT software (v1.1). The intra and inter-assay coefficients of variation for all cytokines tested were < 5 and < 15–20%, respectively.

### Statistical analysis

According to the normality Kolmogorov-Smirnov test, all continuous variables except BMI and age were departed from a normal distribution. Levene test shows that age has not equality of variances. Therefore, non-parametric tests were used for all continuous variables except for BMI and age. Moreover, a correction for not equality of variances was performed for the age variable.

Sociodemographic and clinical variables were analyzed among control and patient groups using Student *T* test, Wilcoxon *W* test or Chi-Square test as necessary. Wilcoxon *W* test was used to compare the basal serum levels of the 21 cytokines between controls and patients in four different analyses as it was mentioned in the study design section. Although these are considered as planned or “a priori” contrasts, as many tests are being conducted, the probability of making a type I error (false positive) is increased. Keeping this in mind, we decided to apply the Bonferroni adjustment for planned comparisons and lowering the threshold of statistical significance to 0.0125 (0.05/4 = 0.0125), being 4 the number of independent contrasts that have been conducted.

For describing the samples, means and standard deviations were used in the case of continuous variables, whereas the total number of observations and percentages were used for qualitative variables. Median serum cytokine levels and interquartile range (IQR) were represented by box plots after performing a natural logarithmic transformation.

STATA 15.0 was used for statistical analysis. Statistical tests were two-tailed with a 99% confidence interval.

## Results

### Differences in sociodemographic and clinical variables

As shown in Table 1, there were no statistically significant differences between healthy volunteers and patients in terms of demographic and clinical variables (all ps *> 0.1*), except for BMI (*t =* − 4.06; *p =* 0.001) in the normal-weight group, and in terms of age (*t =* 2.49; *p* = 0.017) and cannabis-use (*Χ*^2^ = 10.97; *p* = 0.001) in the overweight group.

**Table 1:** Demographic and clinical characteristics between healthy volunteers and patients according to BMI

	Entire simple *N* = 150	Normal-weight Control group *n* = 45	Normal-weight Patient group *n* = 53	Overweight Control group *n* = 30	Overweight Patient group *n* = 22	
	*N* (%)	*n* (%)	*n* (%)	*n* (%)	*n* (%)	Statistics
Sex (female)	76 (50.7)	23 (51.1)	31 (58.5)	13 (43.3)	9 (40.9)	*Χ*^2^ = 0.53; *p* = 0.464^a^ *Χ*^2^ = 0.03 *p* = 0.861^b^
Cannabis users	51 (34.0)	12 (26.7)	24 (45.9)	14 (46.7)	1 (4.6)	*Χ*^2^ = 3.63; *p* = 0.057^a^ *Χ*^2^ = 10.97; *p* = 0.001^b^
Tobacco users^c^	76 (51.4)	22 (48.9)	27 (51.9)	19 (63.3)	8 (38.1)	*Χ*^2^ = 0.09; *p =* 0.766^a^ *Χ*^2^ = 3.16; *p =* 0.076^b^
	Mean (sd)	Mean (sd)	Mean (sd)	Mean (sd)	Mean (sd)	Statistics
Age at onset (years)	29.2 (7.15)	28.2 (6.09)	27.7 (7.33)	29.6 (6.53)	34.5 (7.46)	*t = − 0.33*; *p* = 0.739^a^ *t = 2.49*; *p* = 0.017^b^
BMI at baseline (kg/m^2^)	23.8 (4.59)	22.0 (1.77)	20.3 (2.28)	29.6 (3.83)	28.2 (2.28)	*t = − 4.06*; *p = 0.000*^a^ *t = − 1.50*; *p = 0.139*^b^

^a^Comparison between patients and healthy volunteers in the normal-weight group

^b^Comparison between patients and healthy volunteers in the overweight group

^c^*n* = 148

### Basal serum cytokine levels: analyses stratified by BMI

Out of 75 patients, 53 (70.7%) had a BMI under 25 and thus were considered as normal-weight individuals (BMI mean *=* 20.3; sd *=* 2.28), whereas 22 (29.3%) were considered as overweight individuals (BMI mean *=* 28.2; sd *=* 2.28). Of the control subjects, 45 (60%) were initially considered as normal-weight (BMI mean = 22.0; sd *=* 1.77), whereas 30 (40%) were classified as belonging to the overweight group (BMI mean = 29.6; sd *=* 3.83).

**Fig. 1:**
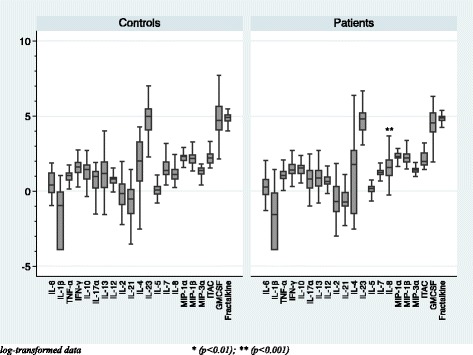
Basal serum cytokine levels in the normal-weight group (BMI < 25)

**Fig. 2:**
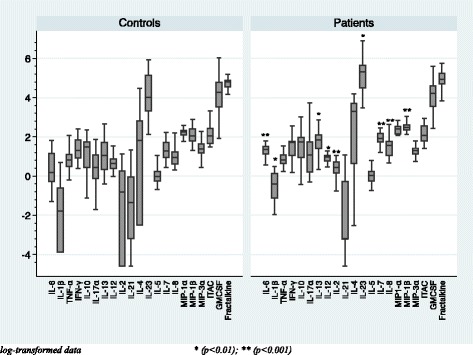
Basal serum cytokine levels in the overweight group (BMI ≥ 25)

**Fig. 3:**
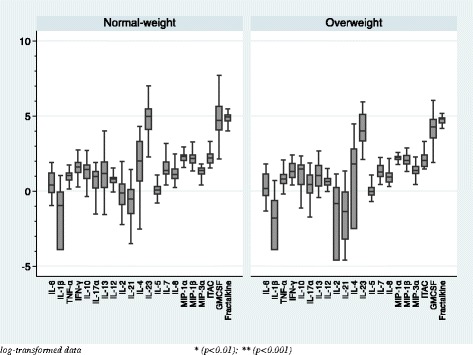
Basal serum cytokine levels within the control group

**Fig. 4:**
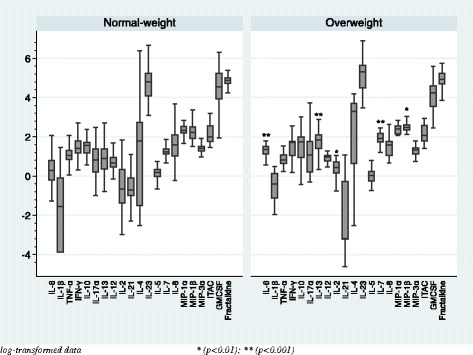
Basal serum cytokine levels within the patient group

In comparison of the basal serum cytokine levels between healthy volunteers and patients with normal weight, Fig. 1 shows that IL-8 was the only cytokine that was higher in patients than in the controls (*p* = 0.001). In comparison of the basal serum cytokine levels between healthy volunteers and patients with overweight as shown in Fig. 2, serum levels of two pro-inflammatory cytokines (IL-6, *p* = 0.000; IL-1β, *p* = 0.003), two chemokines (IL-8, *p* = 0.001; MIP-1β, *p* = 0.001), four Th-1 and Th-2 cytokines (IL-13, *p* = 0.009; IL-2, *p* = 0.001; IL-7, *p* = 0.001; IL-12p70, *p* = 0.010), and one Type-3 cytokine (IL-23, *p* = 0.010) were higher in patients than in the controls. As a secondary analysis, we have compared the basal serum cytokine levels between normal-weight and overweight individuals within both groups of healthy volunteers and patients, as shown in Figs. 3 and 4. No differences were found between the normal-weight and the overweight individuals in the control group (all ps > 0.01), whereas in the patient group, the overweight individuals had higher levels of several cytokines such as IL-6 (*p* = 0.000), IL-13 (*p* = 0.000), IL-2 (*p* = 0.002), IL-7 (*p* = 0.000), and MIP-1β (0.010) than the normal-weight individuals.

## Discussion

These findings support our main hypothesis that excess weight can alter the homeostasis of the immune system and therefore may have an additive pro-inflammatory effect on the one produced by psychosis in the central nervous system (CNS). As far as we know, this is the first research that studies the direct effect of excess weight on basal serum cytokine levels in individuals with FEP.

In the present study, we separated the subjects into two different groups according their BMI (Table 1). In the normal-weight group, only the IL-8 (chemokine) was higher in patients than in healthy volunteers, whereas in the overweight group, we found more differences, as expected: patients had higher levels than the control subjects of two pro-inflammatory cytokines (IL-6 and IL-1β), two chemokines (IL-8, MIP-1β), four Th-1 and Th-2 cytokines (IL-13, IL-2, IL-7, IL-12p70), as well as one Type-3 cytokine (IL-23) (Figs. 1 and 2). IL-8 seems to be the chemokine more related to psychosis and might be considered as a probably state/trait marker of the disease. This is supported by the fact that its levels are not influenced by weight (no differences were found between overweight patients and normal-weight patients, as shown in Fig. 4), as well as its levels are higher in patients than in healthy volunteers in both overweight comparison and in normal-weight comparison (see Figs. 1 and 2). In addition, one article by Hayes et al. (2014) found higher IL-8 levels in the cerebrospinal fluid (CSF) of schizophrenia patients than in healthy volunteers; they concluded that the differences observed were consistent, suggesting these may represent a trait marker associated with psychosis. Regarding the rest of altered cytokines between patients and controls in the overweight group, the cytokines which are more related to overweight are IL-6, TNF-α, and IL-1β. The fact that these cytokines are not higher in the overweight control group than in the normal-weight control group suggest that excess weight in isolation, without the synergistic pro-inflammatory effect of psychosis, might not be enough to alter these cytokines in some cases.

Previous studies carried out in FEP either have excluded the overweight individuals [7, 20, 23] from the analysis or have not considered the BMI in the analysis [6, 18] because the result was similar between patients and controls. Regarding the two articles that did not consider the effect of the BMI, Di Nicola et al. [6] and Noto et al. [18] observed that IL-6, TNF-α, and IL-8 were higher in patients than in healthy volunteers. On the other hand, regarding the three studies which excluded overweight individuals from the analysis, Ding et al. [7] found that IL-6 and IL-17 were increased in patients when compared to controls, Song et al. [23] found that IL-6, TNF-α, and IL-1β were elevated in patients compared to healthy volunteers, and [20] observed that IL-6 and IL-2 were higher in patients than in controls.

We suggest three possible reasons that may explain the discrepancies between our findings in the normal-weight group, in which only IL-8 was increased in patients as opposed to the control subjects, and previous studies that excluded the overweight individuals [7, 20, 23], where several pro-inflammatory cytokines as well as Th-1 and Th-2 cytokines are altered. First, whereas our study population is of Caucasian origin, the studies by Ding et al. [7] and Song et al. [23] were conducted in Asian populations, so some of the differences could be explained by genetic background. Second, cannabis, which is known to play a role in the homeostasis of the immune system [24], has not been considered as a possible confounding variable in the match of any of the three studies. Third, it is important to remark that individuals with identical BMI may have different body compositions regarding fat and muscle, so we cannot exclude the contribution of adipose tissue or of other lymphoid or non-lymphoid cells to the elevated levels of pro-inflammatory cytokines [6, 26].

Excess weight may produce a pro-inflammatory effect when BMI is equal or over 25 [16, 22]. Thus, although there is a statistically significant difference between BMI of healthy volunteers and patients in the normal-weight group (see Table 1), as BMI is under 25 in both subgroups, the difference observed should not assume any effect on the cytokines studied.

In this study, the overweight controls used more cannabis than the overweight patients (*p* = 0.001), as shown in Table 1. Cannabis may have an anti-inflammatory effect [24] that could explain why more differences have been found in the overweight group with regard to the normal-weight group. Thus, we have repeated the previous analysis excluding cannabis-use individuals and found that the results remain similar. Because the cannabis variable is qualitative (consumption yes/no), we may speculate that our findings seem to support the notion that overweight controls might consume small amounts of cannabis and therefore it seems not to have any anti-inflammatory effect on the cytokines studied.

Regarding the likely effect of age in our study, patients in the overweight group are older than the controls (*p* = 0.017), as shown in Table 1. Álvarez-Rodríguez et al. [1] described a positive correlation between age and some circulating pro-inflammatory cytokines, such as TNF-α, IL-1β, IL-6, and IL-12. However, this correlation was observed for individuals with a mean age difference much greater than the one observed in our sample (25 years of age difference VS 5 years of age difference, respectively). In addition, as shown in Fig. 2, patients in the overweight group had higher levels of other cytokines besides the pro-inflammatory ones that have not been previously correlated with age, such as two chemokines (IL-8 and MIP-1β) and also several Th-1 and Th-2 cytokines (IL-13, IL-2, and IL-7).

Three original studies [8, 13, 21] and one meta-analysis [27] have found elevated mRNA expressions of several pro-inflammatory cytokines and Toll-like receptors in post-mortem brains of individuals with schizophrenia. Moreover, as it was mentioned above, Hayes et al. [14] found higher levels of IL-8 in the CSF of psychosis individuals compared to healthy volunteers. However, peripheral blood is a biological matrix not sensitive enough to detect immune disturbances in the CNS. Furthermore, it is possible that CNS immune changes may be only observed after a second hit. Thus, the inflammation induced by the overweight might be the spark that initiates the fire and the inflammation caused by psychosis would be the second hit [16, 22, 25]. The fact that the largest differences in the basal cytokine levels between patients and healthy volunteers were found in the overweight group might be explained by the additive pro-inflammatory effect of both excess weight and psychosis [4, 22]. This is supported by the analyses comparing the basal serum cytokine levels between normal-weight and overweight individuals within the groups of healthy volunteers and patients; Fig. 3 shows no differences in the cytokine levels between overweight and normal-weight healthy volunteers, whereas in Fig. 4, it is depicted that overweight patients have higher inflammatory cytokine and chemokine levels than the normal-weight patients. Keeping this in mind, we suggest that cytokines are probably not the best biomarkers to detect the inflammation that is taking place in the CNS because they are influenced by several confounding factors such as BMI, cannabis use, the taking of analgesics and anti-inflammatory drugs, stress, and age.

This study has several limitations that should be considered: (1) stress level, which is known to influence inflammatory status, was not measured; (2) we have not been able to gauge the possible influence of adipose tissue on the pro-inflammatory cytokines; (3) we were not able to consider the consumption of classical analgesic or anti-inflammatory drugs as possible confounding factors (however, it is important to remark that the subjects from this study are young, healthy individuals without chronic diseases or chronic treatments); and (4) analyses were performed on peripheral blood. We assume that these findings are an indirect reflection of the CNS events; therefore, we would expect to find a greater disturbance of these cytokines in the CNS.

## Conclusions

We found that the most remarkable differences in the basal serum cytokine levels between patients and healthy volunteers were found in the overweight group. In addition, the overweight patients had higher levels of several inflammatory cytokines and chemokines than the normal-weight patients, whereas no differences were found between the normal-weight controls and the overweight controls. Considering these findings, we suggest that excess weight may help to detect immune alterations in peripheral blood that are taking place in the CNS by psychosis, due to a possible additive pro-inflammatory effect that alters the homeostasis of the immune system.

## Abbreviations

BMI: Body mass index
CNS: Central nervous system
CSF: Cerebrospinal fluid
FEP: First-episode psychosis

## References

[1] Álvarez-Rodríguez L, López-Hoyos M, Muñoz-Cacho P, Martínez-Taboada VM (2012). Aging is associated with circulating cytokine dysregulation. Cell Immunol.

[2] Annamalai A, Kosir U, Tek C (2017). Prevalence of obesity and diabetes in patients with schizophrenia. World J Diabetes.

[3] Beumer W, Drexhage RC, De Wit H, Versnel MA, Drexhage HA, Cohen D (2012). Increased level of serum cytokines, chemokines and adipokines in patients with schizophrenia is associated with disease and metabolic syndrome. Psychoneruendocrinology.

[4] Borska L, Kremlacek J, Andrys C, Krejsek J, Hamakova K, Borsky P, Palicka V (2017). Systemic inflammation, oxidative damage to nucleic acids, and metabolic syndrome in the pathogenesis of psoriasis. Int J Mol Sci.

[5] Crespo-Facorro B, Carrasco-Marín E, Pérez-Iglesias R, Pelayo-Terán JM, Fernandez- Prieto L, Leyva-Cobián F, Vázquez-Barquero JL (2008). Interleukin-12 plasma levels in drug-naïve patients with a first episode of psychosis: effects of antipsychotic drugs. Psychiatry Res.

[6] Di Nicola M, Cattaneo A, Hepgul N, Di Forti M, Aitchison KJ, Janiri L, Murray RM, Dazzan P, Pariante CM, Mondelli V (2012). Serum and gene expression profile of cytokines in first-episode psychosis. Brain Behav Immun.

[7] Ding M, Song X, Zhao J, Gao J, Li X, Yang G, Wang X, Harrington A, Fan X, Lv L. Activation of Th17 cells in drug naive, first episode schizophrenia. Prog Neuro-Psychopharmacol Biol Psychiatry. 2014;51:78–82. 10.1016/j.pnpbp.2014.01.001 24447943

[8] Fillman SG, Cloonan N, Catts VS, Miller LC, Wong J, McCrossin T (2013). Increased inflammatory markers identified in the dorsolateral prefrontal cortex of individuals with schizophrenia. Mol Psychiatry.

[9] Fond G, d’Albis MA, Jamain S, Tamouza R, Arango C, Fleischhacker WW, Glenthøj B (2015). The promise of biological markers for treatment response in first-episode psycho-sis: a systematic review. Schizophr Bull.

[10] Fond G, Resseguier N, Schürhoff F, Godin O, Andrianarisoa M, Brunel L, Bulzacka E, et al. Relationships between low-grade peripheral inflammation and psychotropic drugs in schizophrenia: results from the national FACE-SZ cohort. Eur Arch Psychiatry Clin Neurosci. 2017; 10.1007/s00406-017-0847-1. 10.1007/s00406-017-0847-1 29127503

[11] García-Bueno B, Bioque M, Mac-Dowell KS, Barcones MF, Martínez-Cengotitabengoa M, Pina-Camacho L, Rodríguez-Jiménez R, Sáiz PA, Castro C, Lafuente A, Santabárbara J, González-Pinto A, Parellada M, Rubio G, García-Portilla MP, Micó JA, Bernardo M, Leza JC (2014). Pro-/anti-inflammatory dysregulation in patients with first episode of psychosis: toward an integrative inflammatory hypothesis of schizophrenia. Schizophr Bull.

[12] García-Bueno B, Bioque M, MacDowell KS, Santabárbara J, Martínez-Cengotitabengoa M, Moreno C, Sáiz PA, Berrocoso E, Gassó P, Fe Barcones M, González-Pinto A, Parellada M, Bobes J, Micó JA, Bernardo M, Leza JC. Pro-/antiinflammatory dysregulation in early psychosis: results from a 1-year follow-up study. Int J Neuropsychopharmacol. 2014b;18(2) 10.1093/ijnp/pyu037. 10.1093/ijnp/pyu037 PMC4368893 25577666

[13] García-Bueno B, Gassó P, MacDowell KS, Callado LF, Mas S, Bernardo M, Lafuente A, Meana JJ, Leza JC (2016). Evidence of activation of the Toll-like receptor-4 pro-inflammatory pathway in patients with schizophrenia. J Psychiatry Neurosci.

[14] Hayes LN, Severance EG, Leek JT, Gressitt KL, Rohleder C, Coughlin JM, Leweke M, et al. Inflammatory molecular signature associated with infectious agents in psychosis. Schizophr Bull. 2014; p. 963–72. 10.1093/schbul/sbu052. 10.1093/schbul/sbu052 PMC4133679 24743863

[15] Miller BJ, Buckley P, Seabolt W, Mellor A, Kirkpatrick B (2011). Meta-analysis of cytokine alterations in schizophrenia: clinical status and antipsychotic effects. Biol Psychiatry.

[16] Medzhitov R (2008). Origin and physiological roles of inflammation. Nature.

[17] Monteiro R & Azevedo I. Chronic inflammation in obesity and the metabolic syndrome. Mediat Inflamm. 2010; 2010: 289645. 10.1155/2010/289645. 10.1155/2010/289645 PMC2913796 20706689

[18] Noto C, Ota VK, Gouvea ES, Rizzo LB, Spindola LM, Honda PH, Cordeiro Q (2015). Effects of risperidone on cytokine profile in drug-naïve first-episode psychosis. Int J Neuropsychopharmacol.

[19] Pelayo-Terán JM, Pérez-Iglesias R, Ramírez-Bonilla M, González-Blanch C, Martínez-García O, Pardo-García G, Rodríguez-Sánchez JM (2008). Epidemiological factors associated with treated incidence of first-episode non-affective psychosis in Cantabria: insights from the Clinical Programme on Early Phases of Psychosis. Early Interv Psychiatry.

[20] Petrikis P, Voulgari PV, Tzallas AT, Archimandriti DT, Skapinakis P, Mavreas V (2015). Cytokine profile in drug-naive, first episode patients with psychosis. J Psychosom Res.

[21] Rao JS, Kim H-W, Harry GJ, Rapoport SI, Reese EA (2013). Increased neuroinflammatory and arachidonic acid cascade markers, and reduced synaptic proteins, in the postmortem frontal cortex from schizophrenia patients. Schizophr Res.

[22] Schmidt FM, Weschenfelder J, Sander C, Minkwitz J, Thormann J, ChittkaT MR (2015). Inflammatory cytokines in general and central obesity and modulating effects of physical activity. PLoS One.

[23] Song X, Fan X, Li X, Zhang W, Gao J, Zhao J, Harrington A, Ziedonis D, Changes LL (2014). In pro-inflammatory cytokines and body weight during 6-month risperidone treatment in drug naive, first-episode schizophrenia. Psychopharmacology.

[24] Suárez-Pinilla P, López-Gil J, Crespo-Facorro B (2014). Immune system: a possible nexus between cannabinoids and psychosis. Brain Behav Immun.

[25] Šumanović-Glamuzina D, Čulo F, Čulo MI, Konjevoda P, Jerković-Raguž M (2017). A comparison of blood and cerebrospinal fluid cytokines (IL-1β, IL-6, IL-18, TNF-α) in neonates with perinatal hypoxia. Bosn J Basic Med Sci.

[26] Thakore JH, Richards PJ, Reznek RH, Martin A, Dinan TG (1997). Increased intra-abdominal fat deposition in patients with major depressive illness as measured by computed tomography. Biol Psychiatry.

[27] Van Kesteren CF, Gremmels H, de Witte LD, Hol EM, Van Gool AR, Falkai PG, Kahn RS, Sommer IE (2017). Immune involvement in the pathogenesis of schizophrenia: a meta-analysis on postmortem brain studies. Transl Psychiatry.

[28] Yamane H, Paul WE (2012). Cytokines of the γ(c) family control CD4+ T cells differentiation and function. Nat Immunol.

